# Structure-function relationships of the LRRC8 subunits and subdomains of the volume-regulated anion channel (VRAC)

**DOI:** 10.3389/fncel.2022.962714

**Published:** 2022-08-10

**Authors:** Manolia R. Ghouli, Todd A. Fiacco, Devin K. Binder

**Affiliations:** ^1^Division of Biomedical Sciences, School of Medicine, University of California–Riverside, Riverside, CA, United States; ^2^Department of Cell Biology and Neuroscience, Center for Glial-Neuronal Interactions, University of California–Riverside, Riverside, CA, United States

**Keywords:** VRAC, LRRC8A, LRRC8, subdomains, structure, function, pore, subunits

## Abstract

Volume Regulated Anion Channels (VRAC) are critical contributors to cell volume homeostasis and are expressed ubiquitously in all vertebrate cells. VRAC sense increases in cell volume, and act to return cells to baseline volume in a process known as regulatory volume decrease (RVD) through the efflux of anions and organic osmolytes. This review will highlight seminal studies that elucidated the role of VRAC in RVD, their characteristics as a function of subunit specificity, and their clinical relevance in physiology and pathology. VRAC are also known as volume-sensitive outward rectifiers (VSOR) and volume-sensitive organic osmolyte/anion channels (VSOAC). In this review, the term VRAC will be used to refer to this family of channels.

## Introduction

The Volume Regulated Anion Channels (VRAC) are a family of integral membrane channels that are ubiquitously expressed in all vertebrate cells and play a role in regulatory volume decrease (RVD). VRAC were first identified in lymphocytes in 1988 by a group that discovered a novel channel that responded to transmembrane osmotic gradients (such as those generated during cellular swelling events) to induce cellular depolarization, activate K^+^ channels, and subsequently induce the efflux of K^+^, Cl^−^, and water to facilitate RVD (Cahalan and Lewis, 1988). The same year, another group studying intestine 407 human epithelial cells discovered a channel that generated a swelling-activated chloride current in response to hypotonic challenge, and whose inhibition hindered RVD (Hazama and Okada, 1988). Since then, the field has made great advancements in elucidating the identity, structure, function, and clinical relevance of VRAC.

The primary role of VRAC at the cellular level is to maintain volume homeostasis in response to osmotic perturbations. The swelling-induced activation mechanism of VRAC has been an area of contention in the field. Characteristic of cells swollen in response to hypotonic challenge, low intracellular ionic strength modulates the volume sensor for VRAC activation (Cannon et al., 1998; Guizouarn and Motais, 1999; Sabirov et al., 2000). Mild acidification (pH 6) of the cytosolic domain of VRAC was found to enhance channel activity (due to protonation of a group with a pKa of 6.5), however more acidic pH inhibited the channel (due to protonation of a group with a pKa of 6.3), suggesting VRAC operate in a narrow pH window (Sabirov et al., 2000). In nodose neurons, exposure to extracellular acid pH activated VRAC (Wang et al., 2017). This supports the finding that protonation of an extracellular site on these channels increases their single-channel amplitude (due to protonation of a group with a pKa of 4.6; Sabirov et al., 2000). Interestingly, the activation of VRAC due to its sensitivity to acidic pH was found to reduce ischemic neuronal injury, suggesting perturbations to normal physiological pH are detected by VRAC which then facilitate regulatory cellular mechanisms to prevent apoptosis (Wang et al., 2017). Although VRAC display pH-sensitive properties, it is important to note that this family of channels is distinct from the proton-activated chloride channels (ASOR), the cryo-EM structure of which was recently elucidated (Wang et al., 2017). Activation of VRAC conductance depends on the presence of ATP or a nonhydrolyzable ATP analog, and intracellular Ca^2+^ is necessary but not sufficient to activate VRAC (Jackson et al., 1994; Voets et al., 1999; Liu et al., 2019; Centeio et al., 2020). VRAC activity is also affected by constituents of the plasma membrane to which it is tethered. Increased membrane cholesterol content, for example, suppresses VRAC activation in response to mild osmotic gradients. However, it has no effect on the permeability sequence of the anion channel, suggesting plasma membrane rigidity modulates channel open and closed configuration but has little effect on pore conduction properties (Levitan et al., 2000). Cytoskeletal elements and intracellular signaling cascades are also likely to contribute to the VRAC activation mechanism. The rate of VRAC current activation increases with the disruption of F-actin, likely due to decreased tethering of the channel (Schwiebert et al., 1994; Levitan et al., 1995). However, current activation is impaired or altogether inhibited when Rho/Rho-kinase (ROCK) are inhibited (Nilius et al., 1999). Interestingly, ROCK phosphorylates a plethora of substrates including calponin, MARCKS, and EF1α, the cumulative effect of which is an inhibition of F-actin binding (Amano et al., 2010). Thus, it is possible that the disruption of the Rho-Rho kinase pathway leads to an increase in F-actin binding, thereby decreasing VRAC activation. In summary, while the specific details of VRAC activation have yet to be determined, intracellular ionic strength, pH, cytoskeletal and membrane structure and second messengers and signaling pathways are all likely to be important (Nilius et al., 1997, 1998a; Nilius et al., 1999).

Once activated, VRAC mediate RVD through the efflux of anions including the halides fluoride, chloride, bromide, and iodide; larger negatively charged osmolytes including bicarbonate, glutamate, aspartate, glutathione, and lactate; as well as uncharged osmolytes such as taurine, myo-inositol, γ-aminobutyric acid (GABA), and D-serine—secondarily driving water out of the cell (Jackson et al., 1994; Nilius et al., 1998b; Lutter et al., 2017; Friard et al., 2019). VRAC follow an Eisenman type I canonical permeability profile (SCN^−^ > I^−^ > NO_3_^−^ > Br^−^ > Cl^−^ > formate > propionate = methanesulphonate = acetate ≥ F^−^ ≥ butyrate > valerate > gluconate = glucuronate = glutamate; Arreola et al., 1995). Interestingly, although VRAC are primarily known for passive anion efflux, one study found that VRAC can influx the antibiotic Blasticidin S, as well as the essential cancer therapy drugs cisplatin and carboplatin (Planells-Cases et al., 2015). This suggests that some molecules and messengers may travel bidirectionally through this channel.

## Role of VRAC in RVD

Cells actively re-adjust their volume to compensate for transient osmotic challenges *via* regulatory volume decrease (RVD) and regulatory volume increase (RVI). Although RVD involves K^+^ and Cl^−^ efflux and subsequent water loss, the process of RVD is executed by a coalition of channels, transporters, cytoskeletal elements, and intracellular signaling cascades that work in combination. These include but are not limited to various G-protein coupled receptors (GPCRs) such as P2Y_2_ receptors (P2Y_2_Rs) and Ca^2+^-sensing receptors (CaSRs; Hoffmann et al., 2009). Depending on the cell type, the K^+^ channels involved in facilitating RVD may include voltage-gated K^+^ channels, Ca^2+^-gated K^+^ channels, inwardly rectifying K^+^ channels, and two-pore-domain K^+^ channels. Conversely, the swelling-induced efflux of Cl^−^ occurs primarily through VRAC. Other volume-sensitive Cl^−^ channels exist; however, they do not exhibit the combination of mild outward rectification, inactivation at positive membrane potentials, and low field-strength anion permeability of VRAC, making VRAC an optimal channel for RVD (Pasantes-Morales, 2016).

**Figure 1:**
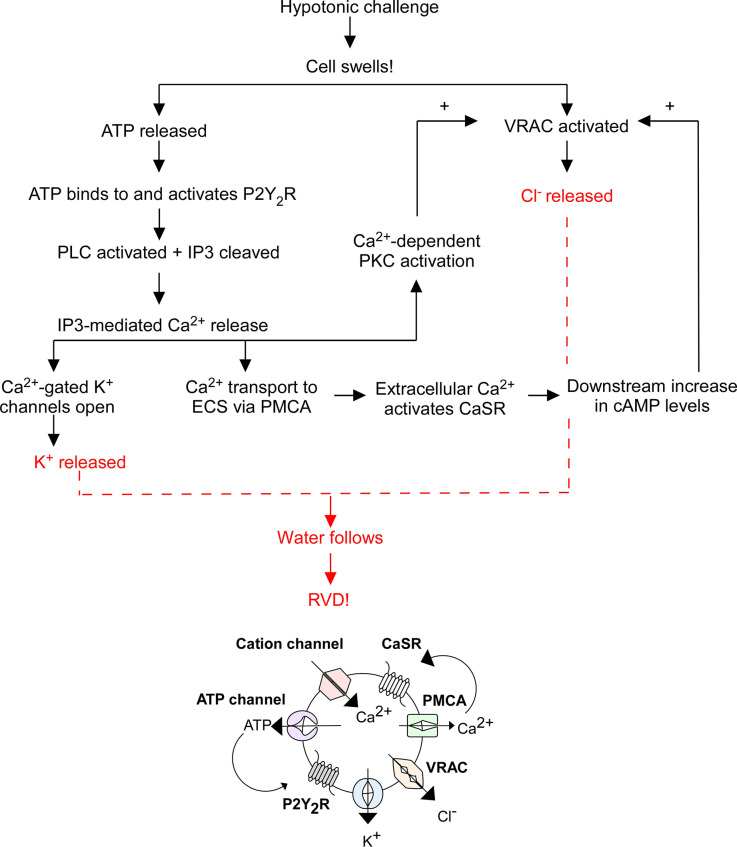
Overview of regulatory volume decrease (RVD). The flowchart above outlines the general mechanism of RVD and is illustrated by the diagram below it. This process includes multiple cellular players including P2Y_2_ receptors (P2Y_2_R), Ca^2+^-sensing receptors (CaSR), plasma membrane Ca^2+^ ATPase (PMCA), cation channels, K^+^ channels, VRAC, phospholipase C (PLC), inositol triphosphate (IP_3_), and cyclic adenosine monophosphate (cAMP), the end result of which is an efflux of K^+^ and Cl^−^ to facilitate the efflux of water and subsequent cell shrinking.

When a cell swells, cation channels open to facilitate Ca^2+^ influx, mechanosensitive ATP-permeable channels release ATP into the extracellular space (ECS), and swell-activated VRAC expel Cl^−^ and other anions. ATP activates P2Y_2_R, thereby increasing IP_3_ levels and stimulating Ca^2+^ release from intracellular stores. Cytosolic Ca^2+^ activates: (1) Ca^2+^-gated K^+^ channels to expel K^+^ from the cell; (2) plasma membrane Ca^2+^ ATPase (PMCA) to send Ca^2+^ into the ECS where it binds to CaSRs to increase cyclic adenosine monophosphate (cAMP) levels, further stimulating VRAC-mediated Cl^−^ release; and (3) Ca^2+^-dependent protein kinases. Specifically, Ca^2+^-dependent protein kinase C (PKC) isoforms α and β were found to play a role in VRAC modulation (Rudkouskaya et al., 2008). ATP is a known potentiator of VRAC activity, however inhibition of PKCα and PKCβ reduced the effect of ATP on VRAC efflux, suggesting the cooperation of PKCα and PKCβ facilitates ATP receptor-dependent VRAC activation (Rudkouskaya et al., 2008). The exact pathway of PKC-mediated VRAC potentiation is unknown; however, it leads to further Cl^−^ release. The compounding efflux of K^+^ and Cl^−^ from the cell (*via* Ca^2+^-gated K^+^ channels and VRAC) triggers RVD by coupled water movement, allowing for cell volume recovery (Figure 1). Hence, VRAC play an important role in RVD through the efflux of Cl^−^ and other anions and organic osmolytes.

VRAC activity may be directly modulated by GPCRs. This is supported by studies showing that treatment of astrocytes in isotonic conditions with ATP resulted in increased VRAC-mediated efflux of small osmolytes (Fisher et al., 2008). This efflux was attenuated using VRAC inhibitors, affirming that ATP modulates VRAC-specific activity and suggesting GPCRs (such as ATP-sensitive P2Y_2_R) may facilitate this effect (Fisher et al., 2008). Interestingly, VRAC activity may also be modified through downstream GPCR-mediated catalyzation of the Rho/ROCK signaling cascade. The inactivation of Rho was found to significantly impair VRAC-mediated Cl^−^ currents (Nilius et al., 1999). This likely occurs due to the downstream effector of Rho/ROCK: myosin light chain (MLC). Inhibition of MLC kinase (MLCK) was found to inhibit VRAC activity, and inhibition of MLC phosphatase (MLCP) was found to potentiate VRAC activity (Nilius et al., 1999). This suggests that GPCRs activate the Rho/ROCK signaling cascade which effects MLCK and MLCP; if MLC is phosphorylated and activated, VRAC is activated and VRAC-mediated RVD may ensue (Nilius et al., 1999).

## VRAC Structure

VRAC are a family of anion channels composed of five LRRC8 subunits (LRRC8A-E), each encoded by its respective LRRC8 gene (*LRRC8A-E*). The LRRC8 subunits assemble in various configurations to yield hexameric transmembrane channel proteins that exhibit a range of biophysical properties depending on their subunit composition (Deneka et al., 2018; Kasuya et al., 2018; Kefauver et al., 2018). Despite the challenge this heterogeneity poses for understanding VRAC morphology, individual LRRC8 subunits (LRRC8A-E) share many structural moieties.

In 2018, three groups independently published the cryo-EM structure of LRRC8A homomer VRAC in digitonin (Deneka et al., 2018; Kasuya et al., 2018; Kefauver et al., 2018). A year later another group generated the cryo-EM structure of LRRC8A homomer VRAC in a lipid nanodisc to better simulate the environment of native channels, and a year after that another group released the cryo-EM structure of LRRC8D homomer VRAC (Deneka et al., 2018; Kasuya et al., 2018; Kefauver et al., 2018; Kern et al., 2019; Nakamura et al., 2020). Although there is no evidence to suggest VRAC exists in the form of homomers in nature, the study of homomers eliminates the complexity and variability observed in the characterization of heteromeric channels, and by the nature of homomeric symmetry it allows for the structural determination of individual LRRC8 subunits.

**Figure 2:**
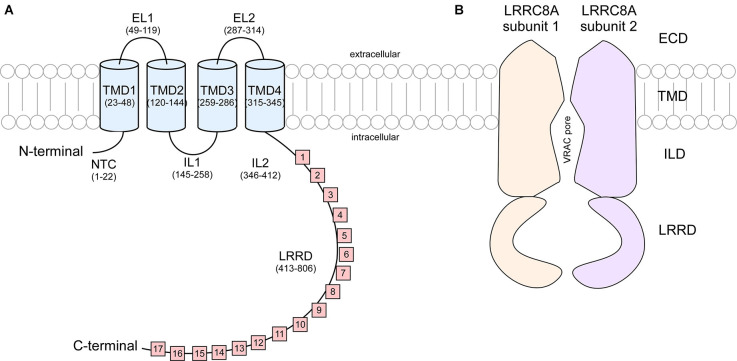
Cartoon representation of LRRC8A subunit structure and cross section of VRAC. **(A)** Each LRRC8A subunit is made up of an intracellular N-terminal coil (NTC, residues 1–22), four transmembrane domains (TMD; residues 23–48, 120–144, 259–286, and 315–345) connected by two extracellular loops (EL, residues 49–119 and 287–314) and two intracellular loops (IL, residues 145–258 and 346–412), followed by 15–17 cytoplasmic leucine rich repeats (residues 413–806; Kasuya et al., 2018). **(B)** Each VRAC is composed of six LRRC8 subunits. This diagram portrays a cross section of an LRRC8A homomer that arranges to produce a hexameric channel with an extracellular domain (ECD), transmembrane domain (TMD). Intracellular linker domain (ILD), and an extensive leucine rich repeat domain (LRRD).

These studies show that LRRC8 isoforms are composed of an intracellular N-terminus defined by a short sequence stretch followed by four transmembrane domains (TMD) and a C terminus linker domain bearing extensive leucine rich repeats (LRR) spanning 15–17 repeats in length (Figure 2). It was originally speculated that the N and C terminus of LRRC8 subunits were extracellular, however Qiu et al. (2014) found that immunohistochemical detection of myc tags located at the terminal ends of the polypeptide sequence was only possible following cell permeabilization, suggesting that these domains were in fact cytosolic (Sawada et al., 2003). LRR domains are evolutionarily conserved protein structures. Because of their role in facilitating receptor-coreceptor communication and their interaction with the toll-like receptors and NOD-like receptors to sense a variety of pathogen-associated molecular patterns and products of avirulence genes, LRR domains contribute to the innate immune response (Ng and Xavier, 2011). This may explain why T-cells and B-cells exhibiting a translocation truncation of the LRRC8A gene progress to agammaglobulinemia, a group of inherited immunodeficiency disorders (Sawada et al., 2003).

**Figure 3:**
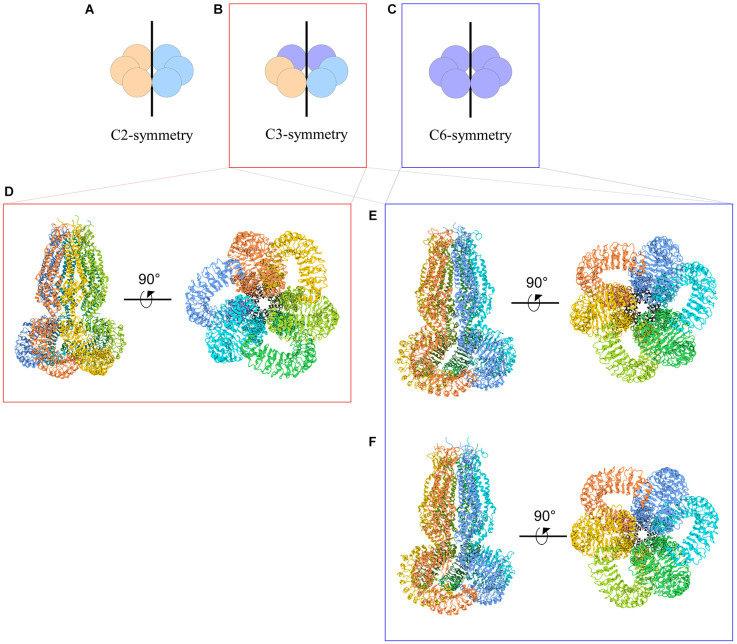
Cartoon representations of LRRC8A homohexameric channels and their symmetries. **(A–C)** Schematic representations of homohexameric channel symmetries. **(A)** C2 symmetry wherein three subunits take on a specific steric conformation (orange), and the other three subunits take on another conformation (blue). This is also referred to as a “dimer of trimers.” **(B)** C3 symmetry wherein two subunits take on the first conformation (orange), two subunits take on a second conformation (blue), and the remaining two subunits take on a third conformation (purple). This is also referred to as a “trimer of dimers.” **(C)** C6 symmetry, wherein each subunit takes on the same conformation. **(D–F)** Cryo-EM structures of LRRC8A homomeric channels. **(D)** Human homohexameric LRRC8A channel (RCSB 5ZSU) at 4.25 Å. C3 symmetry (Kasuya et al., 2018). **(E)** Human homohexameric LRRC8A channel (RCSB 6DJB) at 4.4 Å. C6 symmetry at the ECD, TMD, and ICD; C3 symmetry at the LRRD (Kefauver et al., 2018). **(F)** Mouse analog of homohexameric LRRC8A channel (RCSB 6G9O) at 4.25 Å. C6 symmetry at the ECD, TMD, and ICD; C3 symmetry at the LRRD (Deneka et al., 2018).

The symmetry of VRAC in nature is still an area of uncertainty. Homohexameric channels, such as LRRC8A-homomers, adopt various oligomeric symmetries depending on the steric assembly of the subunits (Figure 3). Through cryo-EM studies, Kefauver et al. (2018) and Deneka et al. (2018) determined that LRRC8A homomers exhibit C6 symmetry at the extracellular, transmembrane, and intracellular domains. However, due to the slight tilt of the LRR domain, the LRRs dimerize into three pairs to exhibit a “trimer of dimers” C3 symmetry. Kevaufer reports a 10° or −20° offset, whereas Deneka suggests a 30°−40° offset of the LRR domain relative to the pore axis. Alternately, Kasuya et al. (2018) argue that LRRC8A homomers exhibit global C3 symmetry despite their reported 22° offset of the LRRs. Interestingly, when the structure of LRRC8A homomers was determined in lipid nanodiscs, the channel exhibited global C6 symmetry (Kern et al., 2019). As for LRRC8D homomers, Nakamura et al. (2020) determined the channels exhibit C2 symmetry. Although these findings are specific to LRRC8A or LRRC8D isomers, the findings are highly relevant to the remaining isomers because LRRC8 subunits share a great deal of sequence homology, with a minimum of 37% (between LRRC8B and LRRC8D) and a maximum of 63% (between LRRC8C and LRRC8E; Abascal and Zardoya, 2012). Thus, insight into the structure-function relationships of the other subunits can be inferred based on their similarities to LRRC8A and LRRC8D structures.

## VRAC and Pannexins

Pannexins are junction proteins in chordates that, like the LRRC8 subunits, are composed of four transmembrane domains that assemble into hexamers. It is postulated that LRRC8 proteins originated from pannexins at the origin of chordates, however under evolutionary constraints the two channels diverged functionally and LRRC8 proteins evolved to specifically express leucine-rich repeat domains (Abascal and Zardoya, 2012). This is supported by the finding that LRRC8 amino domains (short N-terminus stretch and TMDs) share a great deal of homology with pannexins and innexins—non-chordate pannexin paralogs that form gap junctions (Abascal and Zardoya, 2012). Interestingly, LRRC8-like genes were found in the genome of cnidarian *Nematostella vectensis* (a non-chordate), suggesting LRRC8 paralogs may have arisen secondary to horizontal gene transfer in more primitive, non-vertebral and non-chordate organisms (Abascal and Zardoya, 2012). In a comparative analysis, it was found that in addition to the similarity between the four transmembrane helices, subunits of both VRAC and pannexins share sequence homology in the first extracellular loop (EL) just ahead of the second transmembrane helix, and similarly charged residues (arginine, lysine, aspartate and glutamate) reside on the intracellular loop (IL) between TMD2 and TMD3 with low conservation of the sequences in TMD3 and TMD4 relative to the first two TMDs (Abascal and Zardoya, 2012). Both LRRC8 proteins and pannexins lack signal peptides and are trafficked to the plasma membrane through the classical ER-Golgi secretory pathway (Abascal and Zardoya, 2012). The subcellular localization of both LRRC8 proteins and pannexins at the surface of the cell may support their role in facilitating communication between in the intra- and extracellular compartments.

## LRRC8A: The Essential Subunit

Genome-wide silent RNA analysis in HEK293 cells yielded LRRC8A, also known as SWELL1, as the prospective essential subunit of VRAC (Qiu et al., 2014; Voss et al., 2014). Ablation of LRRC8A in HEK293 and HCT116 cells resulted in suppression of swelling-activated VRAC chloride currents, and transfection of *LRRC8A^−/−^* cells with LRRC8A successfully restored currents. Singular ablation of subunits LRRC8B-E did not extinguish the swelling-activated VRAC chloride current to the degree that LRRC8A ablation did (Voss et al., 2014). Concurrent with the previous findings, Qiu et al. (2014) demonstrated robust suppression of swelling-activated VRAC chloride currents following genetic silencing of LRRC8A in HEK293 cells, HeLa cells, and CD4^+^ T lymphocytes. Okada et al. (2017) corroborated these findings in C127 cells. In a separate but similar experiment, LRRC8A was genetically silenced in cortical rat astrocytes using small interfering RNA and when challenged with hypotonic media, siLRRC8A cells yielded reduced VRAC-mediated efflux of taurine and excitatory amino acids glutamate and aspartate (Hyzinski-García et al., 2014). Together, these findings support an essential and necessary role of LRRC8A for VRAC function. Counterintuitively, overexpression of LRRC8A did not increase VRAC conductance but rather decreased the swelling-activated chloride current, suggesting that excess LRRC8A leads to subunit stoichiometry incompatible with normal channel function (Qiu et al., 2014).

The critical role of LRRC8A in VRAC channel function may not be strictly pore-defining. When transfected into HeLa cells, individual LRRC8B-E subunits are retained in the cytoplasm, but when co-transfected with LRRC8A, all subunits localize to the plasma membrane (Voss et al., 2014). These findings were reproduced in HCT116 cells, and suggest that LRRC8A expression is necessary to traffic other LRRC8 subunits to the plasma membrane where they can form viable channels (Yamada and Strange, 2018). Paradoxically, excess expression of LRRC8A may have a dominant-negative action and suppress endogenous LRRC8A from translocating LRRC8B-E to the membrane, thereby inhibiting VRAC currents (Qiu et al., 2014; Voss et al., 2014; Syeda et al., 2016; Okada et al., 2017). The truncation of the LRRC8A C-terminus in the case of agammaglobulinemia leads to cytoplasmic retention of the subunit, suggesting that the LRR domain of LRRC8A facilitates subunit trafficking (Voss et al., 2014).

Importantly, LRRC8A homomers (*LRRC8B/C/D/E^−/−^*) in HeLa cells did not produce VRAC chloride currents when measured by whole-cell recording (Syeda et al., 2016). However, when the LRRC8A homomers were transferred from HeLa cells into a lipid bilayer, DCPIB-sensitive, hypotonicity-induced currents were restored (Syeda et al., 2016). At face value, discrepancy in these findings appears to depend on the experimental model; however, Kefauver et al. (2018) later found DCPIB-sensitive, hypotonicity-induced currents in LRRC8A homomers in the same conditions as those used previously by Syeda et al. (2016). These conflicting data warrant further investigation to resolve whether LRRC8A homomers (*LRRC8B/C/D/E^−/−^*) can form functional channels in native cells.

## LRRC8B-E: Function-Refining Subunits

Swell-activated VRAC chloride currents were abolished in quintuple LRRC8 KOs (*LRRC8^−/−^*) wherein all five LRRC8 subunits (LRRC8A-E) were ablated from HCT116 and HEK293 cells (Voss et al., 2014). Transfection of *LRRC8*^−/−^ cells with LRRC8A alone did not restore the chloride current. Instead, Voss et al. (2014) found that LRRC8A required co-transfection with either LRRC8C or LRRC8E to restore VRAC current to near-native levels. Co-transfection of LRRC8A with LRRC8D only partially restored the VRAC current, while co-transfection with LRRC8B failed to restore the current altogether. Conversely, Syeda et al. (2016) reported that *LRRC8B/C/E^−/−^* and *LRRC8B/C/D^−/−^* channels (the equivalent of LRRC8A/D and LRRC8A/E channels) exhibited current comparable to wild type (WT), whereas *LRRC8B/D/E^−/−^* channels (the equivalent of LRRC8A/C channels) exhibited approximately half the current of WT (Syeda et al., 2016). Thus, the findings of Voss et al. (2014) suggest that LRRC8A must be co-transfected with either LRRC8C or LRRC8E to restore native VRAC current, whereas Syeda et al. (2016) suggest LRRC8A must exist as a heteromer with either LRRC8D or LRRC8E to generate native-like currents. The discrepancies between these findings are not negligible and highlight the need for further study to ascertain the influence of subunit composition on heteromeric VRAC activity.

### LRRC8B

LRRC8B is most highly expressed in the brain, followed by an intermediate level of expression in the kidneys and lung, and is altogether undetectable in the spleen (Pervaiz et al., 2019). Overexpression of this subunit in HEK293 cells was found to reduce ER-mediated Ca^2+^ release, and reciprocally, LRRC8B-knockout cells exhibit slower depletion of Ca^2+^ stores in the ER (Ghosh et al., 2017). It is possible that without co-expression of LRRC8A, LRRC8B homomers in the ER form leaky Cl^−^ channels that secondarily participate in Ca^2+^ homeostasis, whereas LRRC8B in the presence of LRRC8A localizes to the plasma membrane (with at least one other LRRC8 paralog) to form functional VRAC (Ghosh et al., 2017).

### LRRC8C

The highest level of LRRC8C expression is in the heart, where this subunit is the most abundant LRRC8 paralog (Pervaiz et al., 2019). In heteromeric complexes with LRRC8A and LRRC8E, LRRC8C acts as a broadly expressed 2’3’-cyclic-GMP-AMP (cGAMP) transporter (Lahey et al., 2020). Cyclic GMP, an immunotransmitter, is released from diseased cells and taken up by host cells to initiate the immune STING response (Decout et al., 2021). Additionally, LRRC8C in complex with LRRC8A associate with NADPH oxidase 1 (Nox1) to regulate superoxide production and activation of tumor necrosis factor-α (TNFα) to potentiate inflammatory responses (Choi et al., 2021). These findings point to the role of LRRC8C-containing VRAC in mediating multiple immune processes.

### LRRC8D

LRRC8D is expressed at similar levels across organ systems (Pervaiz et al., 2019). Unlike LRRC8C and LRRC8E, LRRC8D-containing VRAC complexes inhibit cGAMP transport (Lahey et al., 2020). However, the LRRC8D subunit is required for the import of antibiotic blasticidin S, and its presence with LRRC8A is required for 50% of the uptake of anti-cancer drug cisplatin (Lee et al., 2014; Planells-Cases et al., 2015). This highlights the role of LRRC8D-bearing VRAC in the bidirectional transport of therapeutics. Although VRAC are mainly known for their role in anion efflux, LRRC8D-containing VRAC are also permeable to neutral and positively-charged compounds such as myo-inositol, taurine, GABA, and lysine (Lutter et al., 2017). In the kidney, LRRC8D is predominantly expressed in the proximal tubule, and constitutive deletion of this subunit results in proximal tubular injury, increased diuresis, and Fanconi-like symptoms (López-Cayuqueo et al., 2022). This suggests that LRRC8D (coupled with LRRC8A) form channels that are responsible for the trafficking of various metabolites, and disruption of LRRC8D precipitates the accumulation of inflammatory molecules.

### LRRC8E

The highest levels of LRRC8E expression are found in the lung and spleen, where this subunit is expressed at similar levels to LRRC8A. As mentioned above, LRRC8E forms heteromeric complexes with LRRC8A and LRRC8C to transport cGAMP to initiate immune activity (Lahey et al., 2020). Additionally, when LRRC8A/E heteromers are challenged with oxidative agents such as chloramine-T or *tert*-butyl hydroperoxide, their activity increases greater than 10-fold, whereas LRRC8A/C and LRRC8A/D heteromers are inhibited by reactive oxygen species (ROS), suggesting that VRAC containing LRRC8E are involved in the mediation of ROS induced pathologies (Gradogna et al., 2017). Interestingly, LRRC8E-bearing VRAC are most permeable to negatively charged compounds such as aspartate (Lutter et al., 2017).

## Structure-Function Relationships of VRAC Subunit Subdomains

As highlighted above, not all VRAC pores are built the same. Any combination of LRRC8 subunits will yield nuances in channel permeability and conductivity. Even among VRAC composed of the same subunits, questions remain about how various subunit ratios and arrangements alter the biomechanics of the channel. Cryo-EM studies conducted within the past three years have provided great insight into how VRAC function is defined by subunit subdomains (Deneka et al., 2018; Kasuya et al., 2018; Kefauver et al., 2018; Kern et al., 2019; Nakamura et al., 2020). Study and characterization of LRRC8A and LRRC8D homomers has provided a simplified, symmetrical analysis of channel structure, enabling inferences to be made about properties of native VRAC. Each VRAC pore is defined by narrowing constrictions found at the first extracellular loop (EL1) of the extracellular domain (ECD), the first transmembrane domain (TMD1), the N-terminal coil (NTC), and the C-terminal structures.

### The pore at EL1 of the ECD

The narrowest part of the VRAC pore exists at the ECD of the channel. In LRRC8A homomers, approximately 25 Å above the membrane surface each of the LRRC8A subunits projects an arginine (R103) bulky side chain into the pore lumen to form a “ring of arginines” that constricts the pore to a diameter of 5.8–7.6 Å (Deneka et al., 2018; Kefauver et al., 2018; Kern et al., 2019). At the equivalent position in LRRC8D homomers, each subunit projects the aromatic ring of a phenylalanine residue (F143) into the pore lumen to constrict the pore to a diameter of 11.5 Å (Nakamura et al., 2020). The structure of the pores of other LRRC8 homomers has not yet been elucidated by cryo-EM. Because the pore of LRRC8D homomers is wider than that of LRRC8A homomers, it is possible that inclusion of LRRC8D subunits in heteromeric VRAC leads to increased channel permeability (Nakamura et al., 2020).

To determine whether the charge of the residues at the ECD channel narrowing dictates subunit-specific pore properties, positively-charged R103 in LRRC8A was mutated into uncharged phenylalanine (R103F) and co-transfected with LRRC8C (LRRC8A(R103F)/C) into *LRRC8^−/−^* HeLa cells. This resulted in significantly reduced reversal potential of VRAC currents compared to control LRRC8A/C channels (Kefauver et al., 2018). Mutant LRRC8A (R103A)/C channels also displayed increased permeability to Na^+^ (Deneka et al., 2018). Conversely, uncharged F143 in LRRC8D subunits was mutated into a positively-charged arginine and co-transfected with LRRC8A (LRRC8A/D(F143R)) into *LRRC8^−/−^* cells. Compared to control LRRC8A/D channels, the mutant heteromeric channels exhibited decreased permeability to glutamate and gluconate, and where control channels exhibited higher selectivity for glutamate over gluconate, mutant channels showed no preference between the two (Nakamura et al., 2020). These findings indicate that the residues lining the narrowest region of the pore in both LRRC8A and LRRC8D subunits sterically influence the pore, thereby defining the anion selectivity of these channels.

Point mutations of K98 and D100 in LRRC8A (and equivalent residues of LRRC8C and LRRC8E)—amino acid residues lining the end of the first extracellular loop—caused significant changes in VRAC voltage-dependent inactivation kinetics and reduced the I^−^ > Cl^−^ selectivity (P_I_/P_Cl_ = 1.25 in WT, P_I_/P_Cl_ =1.12 in K98E; Ullrich et al., 2016). Based on findings from point mutations in the ECDs, homomeric chimeras were used to evaluate the role of longer peptide stretches in this domain. A sequence of the first extracellular loop (EL1) of each LRRC8A subunit in LRRC8A homomers was replaced by the equivalent EL1 sequence of LRRC8C, LRRC8D, and LRRC8E. The chimeric homomer LRRC8A-8C(EL1) exhibited normal swell-activated VRAC conductance compared to LRRC8A/C heteromers, while LRRC8A-8E(EL1) generated small constitutively-active outwardly rectifying currents that were not activated by cell swelling but were inactivated by cell shrinking compared to LRRC8A/E channels. Last, LRRC8A-8D(EL1) did not generate measurable currents compared to LRRC8A/D channels (Yamada and Strange, 2018). These findings suggested that peptide stretches in the EL of LRRC8C (but not LRRC8E or LRRC8D) may serve as critical components of the VRAC pore. Together, these findings demonstrate that the subunit-specific amino acid composition and peptide stretches lining the ECD influence the size and charge selectivity of ions, as well as channel activity of VRAC.

### The pore at TMD1

The first TMD of each subunit marks the second narrowing of the VRAC pore. In LRRC8A homomers, two polar uncharged threonine residues at positions 44 and 48 of each subunit constrict the pore to a width of 13.7 Å (Deneka et al., 2018). Cysteine replacement of T44 (T44C) increased the I^−^ > Cl^−^ selectivity (P_I_/P_Cl_ = ~1.3 in WT, P_I_/P_Cl_ = 1.59 in T44C; Qiu et al., 2014). Application of 2-sulfonatoethyl methanethiosulfonate (MTSES), a negatively-charged covalent modifying agent of cysteine residues, to mutant channels significantly reduced channel activity, suggesting that T44 defines the pore constriction (Qiu et al., 2014). Glutamate replacement of T44 (T44E) produced currents comparable to control channel currents (P_I_/P_Cl_ = 1.27 in T44E), whereas arginine replacement (T44R) significantly reduced I^−^ > Cl^−^ selectivity of mutant channels (P_I_/P_Cl_ = 1.15 in T44R), suggesting that the identity of residue 44 of the LRRC8A subunit contributes to VRAC ion selectivity (Qiu et al., 2014). Although too wide to coordinate the movement of dehydrated ions, this region may still facilitate water-solvated ion movement (Deneka et al., 2018). In LRRC8D homomers, T48 at the TMD is responsible for reducing the pore diameter to 19 Å, slightly wider than that of LRRC8A homomers.

### The pore at the NTC

Another constriction of the LRRC8D homomer occurs specifically at the level of the N-terminus (Nakamura et al., 2020). The N-termini of LRRC8 isomers are composed of a short peptide stretch of approximately 18 amino acid residues whose 3D structure has not yet been determined due to functional limitations of cryo-EM on flexible regions. This short stretch of peptides is reported to form an N-terminal coil (NTC) that lies parallel to the plasma membrane’s inner leaflet and projects into the pore lumen (Deneka et al., 2018; Kasuya et al., 2018; Kefauver et al., 2018). In innexins, channels that share a great deal of homology with VRAC, the NTC forms a pore funnel that marks the site of greatest steric constriction, suggesting the NTC of VRAC may play a similar role (Kefauver et al., 2018). In LRRC8D homomers, the 3D structure of the NTC is not completely resolved. However, it was determined that three residues of the NTC, Leu-4, Val-7, and Leu-10, face the pore. When mutated to cysteines, treated with MTSES, and co-transfected with LRRC8A into *LRRC8^−/−^* cells, LRRC8A/D(V7C) and LRRC8A/D(L10C) mutant channels exhibited decreased swell-induced VRAC currents whereas LRRC8A/D(L4C) showed increased current, which indicates these residues face the pore lumen and influence activity of the channel (Nakamura et al., 2020).

Due to the incompletely resolved 3D structure of the extreme N-terminus, functional assays were conducted to determine the influence of specific N-terminal residues on VRAC properties. Across LRRC8 isomers, residues Glu-6 (E6) and Gln-14 (Q14) are conserved, and the other NTC residues exhibit great homology (Zhou et al., 2018). Zhou et al. (2018) conducted a series of mutagenesis studies using LRRC8A/C heteromeric channels. N-terminal residues (positions 2–14) of either one or both isomers were replaced with cysteine, and the single (only LRRC8A) or double (LRRC8A and LRRC8C)-mutant channels were treated with MTSES to evaluate residue-specific changes to channel conductance. The results indicated that single or double mutations to positions 2–4, as well as double mutations to positions 5 and 7, completely abolished VRAC Cl^−^ currents (Zhou et al., 2018). Mutations to position 6 increased I^−^ > Cl^−^ selectivity (P_I_/P_Cl_ = 1.29 in WT, P_I_/P_Cl_ = 2.29 in E6C; Zhou et al., 2018). Conjugation of cysteine mutants with Cd^2+^ at positions 6, 8, and 9 abolished VRAC Cl^−^ currents (Zhou et al., 2018). Cd^2+^ is a metal ion that coordinates cysteine residues within 5–7 Å of one another, suggesting that N-terminal residues at positions 6, 8, and 9 are close enough to the conduction pathway that their coordination by Cd^2+^ would inhibit channel currents (Jalilehvand et al., 2009). Interestingly, another group determined that singular, as opposed to double, cysteine replacement mutation of position 5 coupled with MTSES application was sufficient to suppress whole-cell current recordings (Kefauver et al., 2018). Together, these findings suggest the extreme N-terminus residues of LRRC8 subunits fold back into the ion translocation pathway, interacting structurally and electrostatically to form another narrowing of the pore to further refine the size and charge selectivity of VRAC (Zhou et al., 2018).

### The pore at C-terminal structures

The last major constrictions of the VRAC pore occur at the cytosolic intracellular loop (IL) and the LRR domain of the C-terminus. The IL comprises a short peptide stretch that connects TMDs 2 and 3. Homomers of LRRC8C, LRRC8D, or LRRC8E form non-functional channels due to lack of the essential VRAC subunit LRRC8A. When the intracellular loop (IL) of LRRC8C, LRRC8D, and LRRC8E was replaced with the 25-amino acid IL sequence of LRRC8A (residues D182-E206), the respective chimeric homomers (LRRC8C-8A(IL), LRRC8D-8A(IL), and LRRC8E-8A(IL)) exhibited normal volume-dependent VRAC activity (Yamada and Strange, 2018). This suggests that the IL domain of LRRC8A may be key to its role as the essential VRAC subunit.

Cryo-EM studies found that the LRRC8A isomer has 15–17 LRRs at the extreme C-terminus, and similarly the LRRC8D isomer has 15 LRRs (Deneka et al., 2018; Kasuya et al., 2018; Kefauver et al., 2018; Kern et al., 2019; Nakamura et al., 2020). LRRs form long twisting arches spanning 80 Å in length, and according to various accounts the LRRs in LRRC8A isomers are oriented about either a C3 or C6 symmetrical axis, and in LRRC8D isomers LRRs are oriented about a C2 symmetrical axis. In the case of C3 symmetry, LRRC8A LRRs are reported to arrange in such a way that 12 Å wide fenestrations develop at the interface of the domain pairs, rendering the LRR domain of VRAC as a pore defining structure (Deneka et al., 2018). However, another group reported that the fenestrations of LRRC8A isomers are larger, approximately 35–40 Å in width, allowing for ions and osmolytes to readily pass through (Kefauver et al., 2018). The cryo-EM study conducted with the LRRC8D homomers concluded that the heterogeneity of LRR domain configuration and fenestration size can be attributed to the flexibility of the LRR domains as a function of compact and relaxed channel conformations (Nakamura et al., 2020).

The truncation of the two terminal LRRs of LRRC8A was reported in a patient with agammaglobulinemia, an inherited disease of immune deficiency, who exhibited abnormal T and B cell development (Platt et al., 2017). In an experiment inspired by this finding, the 15 LRRs of LRRC8A were truncated, leading to dramatically diminished VRAC Cl^−^ currents, suggesting that the LRR domains of LRRC8A may be critical to the gatekeeping of the VRAC pore from the cytosolic side of the channel (Platt et al., 2017). Truncation of the C-terminus of LRRC8A as in patients with agammaglobulinemia caused cytoplasmic retention of all LRRC8 subunits, suggesting that the LRRD of LRRC8A is essential to the trafficking of this subunit, and therefore the remaining subunits, to the plasma membrane (Voss et al., 2014). This may account for the diminished VRAC current observed.

Deneka et al. (2018) previously determined that LRRC8A homomers exhibit C6 symmetry about the pore, but due to an angular offset, the LRR domains exhibit C3 symmetry. To further evaluate the role of the LRR domain of the essential subunit LRRC8A in channel activation, five synthetic nanobodies termed sybodies (Sb1–5) were used to target specific epitopes of the cytoplasmic domain (Deneka et al., 2021). Tightly interacting LRR domains were defined as left (l) or right (r) subunits, according to their dimerization as viewed from the extracellular face. The structures of allosterically modified VRAC (LRRC8A-Sb complexes) were generated by cryo-EM, and tightly interacting LRR domains were defined as left (l) or right (r) subunits according to their dimerization as viewed from the extracellular face. Sybodies Sb1, Sb2, and Sb3 targeted epitopes on the convex surface of all LRR domains to inhibit VRAC function, whereas Sb4 and Sb5 targeted epitopes on the concave surface of only r subunits of the channel to potentiate VRAC activity (Deneka et al., 2021). This could imply that allosteric modifiers of VRAC that bind to the convex surface of LRRs reduce flexibility of the cytosolic domains, thereby stabilizing a closed channel conformation, whereas allosteric modifiers that bind the concave surface of LRRs increase mobility of the domain, thereby stabilizing an open channel conformation (Deneka et al., 2021).

**Figure 4:**
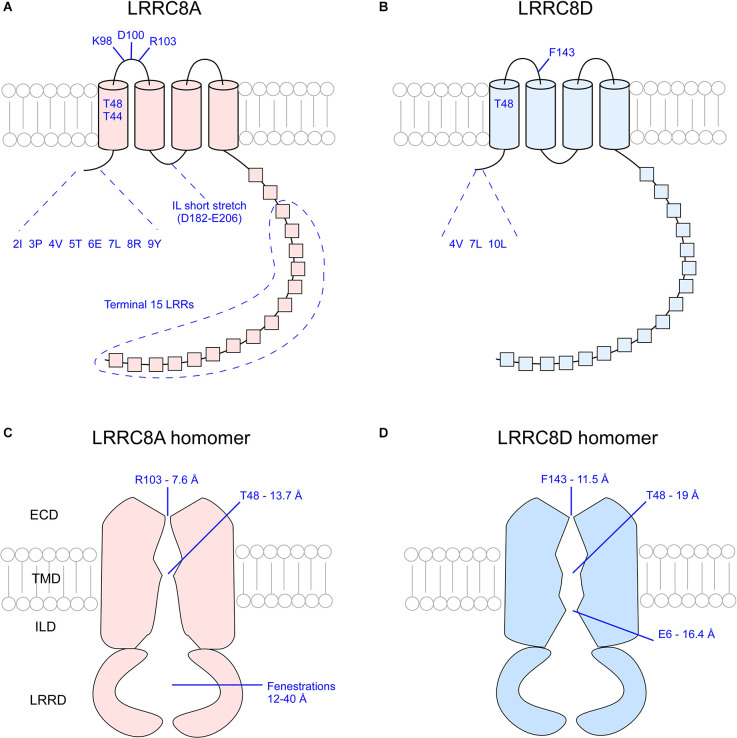
Overview of critical pore-defining amino acid residues of the LRRC8A and LRRC8D subunits as determined by cryo-EM and functional analysis experiments. Critical residues of singular LRRC8A **(A)** and LRRC8D **(B)** subunits. Visualization of the constrictions of the pores of LRRC8A homomers **(C)** and LRRC8D homomers **(D)**. ECD, extracellular domain; TMD, transmembrane domain; ILD, intracellular linker domain; IL, intracellular loop; LRR, leucine rich repeat; LRRD, leucine rich repeat domain.

Taken together, studies to date paint a picture of a VRAC channel with six LRRC8 subunits that assemble to form a funnel-like pore with multiple narrow pore-defining regions (Figure 4). The permeability and gating of VRAC depend on specific residues and sequence stretches at the ECD, the TMD, and the IL connecting TMD2 and TMD3, as well as variable influence by the NTC and LRR fenestrations on the cytoplasmic face of the pore. Although the LRRC8 isomers share homologous sequences, there remains variability among them. Additional studies are needed to determine how different combinations of LRRC8B-E with LRRC8A may impact channel form and function.

## Summary

Volume-regulated anion channels (VRAC) are a heterogenous family of membrane channels that play a critical role in volume homeostasis in vertebrate cells. In conditions of cell swelling, VRAC mediate the efflux of anions and organic osmolytes to coordinate cell volume recovery. The permeability and selectivity of each VRAC channel is a product of its specific subunit composition. Abundant evidence suggests that the LRRC8 gene family encodes the pore-defining components of VRAC. However, how their interactions define channel gating mechanisms and selective permeability remain to be elucidated. In this review, we highlighted seminal studies that improved understanding of VRAC channel structure and function.

VRAC have the potential to be valuable targets for drug discovery. Therapies that target the localization of VRAC in B cells to the cell membrane or therapies that activate VRAC in these cells may curb the immunodeficiencies seen in patients with agammaglobulinemia. Similarly, therapies that work to upregulate VRAC in the trabecular meshwork of the eyes may increase aqueous outflow thereby decreasing intraocular pressure in patients with glaucoma. Conversely, therapies that inhibit or downregulate VRAC may work to diminish the effects of excitotoxicity seen in stroke. VRAC may also serve as a target for cancer treatment due to its permeability to cancer therapies.

In 2019, Yang et al. (2019) found that deletion of LRRC8A in mGFAP^+^ cells played a protective role by attenuating glutamate neurotoxicity in a model of stroke. This finding has far-reaching implications regarding the targeting of VRAC to improve clinical outcome in instances of acute or chronic cellular edema, including stroke, hydrocephalus, water intoxication, epilepsy, and other conditions of neuroinflammation. A VRAC-specific antagonist could theoretically decrease the release of glutamate from glutamate-handling cells like astrocytes, thereby curbing excitotoxicity in neurodegenerative disease and disorders (Zhang et al., 2008). Similarly, a VRAC antagonist targeting ependymal cells lining the ventricles in the brain could restrict the release of fluid from these cells (decreased RVD), thereby slowing the development of hydrocephalus—a disease caused by the buildup of cerebrospinal fluid in the ventricles. On the other hand, a VRAC agonist could quicken RVD in cases in which returning swollen cells to baseline volume is favorable, such as in acute and chronic cellular edema. Thus, restoration of volume homeostasis *via* VRAC modulation by agonists or antagonists may offer a novel therapeutic strategy in multiple diseases.

## Author Contributions

MG made substantial contributions to the design drafting of the article. TF and DB made substantial contributions to the critical review of the article. All authors contributed to the article and approved the submitted version.

## Conflict of Interest

The authors declare that the research was conducted in the absence of any commercial or financial relationships that could be construed as a potential conflict of interest.

## Publisher’s Note

All claims expressed in this article are solely those of the authors and do not necessarily represent those of their affiliated organizations, or those of the publisher, the editors and the reviewers. Any product that may be evaluated in this article, or claim that may be made by its manufacturer, is not guaranteed or endorsed by the publisher.
